# Phyto-Enrichment of Yogurt to Control Hypercholesterolemia: A Functional Approach

**DOI:** 10.3390/molecules27113479

**Published:** 2022-05-28

**Authors:** Harsh Kumar, Kanchan Bhardwaj, Natália Cruz-Martins, Ruchi Sharma, Shahida Anusha Siddiqui, Daljeet Singh Dhanjal, Reena Singh, Chirag Chopra, Adriana Dantas, Rachna Verma, Noura S. Dosoky, Dinesh Kumar

**Affiliations:** 1 School of Bioengineering & Food Technology, Shoolini University of Biotechnology and Management Sciences, Solan 173229, Himachal Pradesh, India; microharshs@gmail.com (H.K.); mails4sharmaruchi@gmail.com (R.S.); adrianadantas@shooliniuniversity.com (A.D.); 2 School of Biological and Environmental Sciences, Shoolini University of Biotechnology and Management Sciences, Solan 173229, Himachal Pradesh, India; kanchankannu1992@gmail.com (K.B.); rachnac83@gmail.com (R.V.); 3 Department of Biomedicine, Faculty of Medicine, University of Porto, 4200-319 Porto, Portugal; 4 Institute for Research and Innovation in Health (i3S), University of Porto, 4200-135 Porto, Portugal; 5 Institute of Research and Advanced Training in Health Sciences and Technologies (CESPU), Rua Central de Gandra 1317, 4585-116 Gandra PRD, Portugal; 6 TOXRUN-Toxicology Research Unit, University Institute of Health Sciences, CESPU, CRL, 4585-116 Gandra PRD, Portugal; 7 German Institute of Food Technologies (DIL e.V.), Prof.-von-Klitzing Str. 7, 49610 D-Quakenbrück, Germany; s.siddiqui@dil-ev.de; 8 Department of Biotechnology and Sustainability, Technical University of Munich, Schulgasse 22, 94315 Straubing, Germany; 9 School of Bioengineering and Biosciences, Lovely Professional University, Phagwara 144411, Punjab, India; daljeetdhanjal92@gmail.com (D.S.D.); reena.19408@lpu.co.in (R.S.); chirag.18298@lpu.co.in (C.C.); 10 Aromatic Plant Research Center, 230 N 1200 E, Suite 100, Lehi, UT 84043, USA

**Keywords:** functional foods, yogurt, phyto-enrichment, hypercholesterolemia

## Abstract

Cholesterol is essential for normal human health, but elevations in its serum levels have led to the development of various complications, including hypercholesterolemia (HC). Cholesterol accumulation in blood circulation formsplaques on artery walls and worsens the individuals’ health. To overcome this complication, different pharmacological and non-pharmacological approaches are employed to reduce elevated blood cholesterol levels. Atorvastatin and rosuvastatin are the most commonly used drugs, but their prolonged use leads to several acute side effects. In recent decades, the potential benefit of ingesting yogurt on lipid profile has attracted the interest of researchers and medical professionals worldwide. This review aims to give an overview of the current knowledge about HC and the different therapeutic approaches. It also discusses the health benefits of yogurt consumption and highlights the overlooked phyto-enrichment option to enhance the yogurt’s quality. Finally, clinical studies using different phyto-enriched yogurts for HC management are also reviewed. Yogurt has a rich nutritional value, but its processing degrades the content of minerals, vitamins, and other vital constituents with beneficial health effects. The option of enriching yogurt with phytoconstituents has drawn a lot of attention. Different pre-clinical and clinical studies have provided new insights on their benefits on gut microbiota and human health. Thus, the yogurtphyto-enrichment with stanol and β-glucan have opened new paths in functional food industries and found healthy andeffective alternatives for HC all along with conventional treatment approaches.

## 1. Introduction

Low-density lipoprotein (LDL) cholesterol and serum total cholesterol (TC) levels are both linked to a high risk of ischemic heart disease found in people at a young age and people at low risk of coronary heart disease [1,2,3,4]. With the increasing incidence of hypercholesterolemia and associated cardiovascular risk, safe, effective, and inexpensive therapeutic approaches were developed to manage such affections [5]. However, over time, it is necessary to understand the extent of blood lipid-induced complications and how they can be managed, which will allow a clear understanding of coronary risk factors among populations [4,5,6,7]. Such studies will also help to identify opportunities to reduce coronary heart risk [8]. According to the World Health Organization (WHO) guidelines, in Kazakhstan, the age-standardized prevalence of total cholesterol increase (≥6.2 mmol/L) was estimated at 12%, which is close to the Russian Federation (15%), but much higher than other Central Asian countries. For instance, Turkmenistan estimated the age-standardized prevalence of total cholesterol increase as the highest, i.e., 8%, and the minimum was 5% in Tajikistan. However, in Central Asian countries, it was estimated to be much lower than in other developed countries, such as Germany (25%) and England (22%) [9].

Regarding blood cholesterol management and distribution, national population-based studies have been conducted in some countries [10,11,12,13,14,15]. From 1999 to 2003, hypercholesterolemia (HC) in Western Europe contributed to heart attacks by about 45%, and in Central and Eastern Europe, it accounted for approximately 35% of heart attack cases [16,17]. However, the risk of heart attack is three times greater in people with high cholesterol levels than those with normal blood lipid profiles. WHO has also demarcated some unhealthy diets, leading to increased cardiovascular risk, e.g., free sugar, high-fat diets, salt, and sugars low in complex carbohydrates [18].

Nowadays, functional foods are referred to as “superfoods”, and, when consumed as part of the daily diets, are believed to have the potential to reduce the risk of several diseases [19]. There are a variety of food products amid the superfoods, including fruits, vegetables, nuts, seeds, and dairy products such as kefir and yogurt [20].

Milk and its products represent an essential part of a healthy balanced diet, rich in proteins and micronutrients [21,22]. Nonetheless, there was a misconception regarding dairy products consumption in the past, linked to the premise that they are high-risk factors for cardiovascular diseases and create adverse effects on human health due to the presence of a marked content of saturated fatty acids [23]. Though, this claim was proved wrong by the fact that dairy products have functional components (calcium, milk proteins, and phospholipids) of high nutritional value, which are efficient in lowering the risk of cardiovascular disease. Their health benefit is attributed to their probiotic effects and the ability to modulate the lipoproteins metabolism [24,25]. More recently, studies have even reported that the consumption of dairy products does not cause any cardiovascular disease [21,22,23,26]. For example, yogurt, a fermented milk product, has been reported to have nutrients with beneficial effects on glucose metabolism, lipid metabolism, and obesity [21,27,28,29,30,31,32]. Considering these aspects, the fortification of yogurt with phytoconstituents may be viewed as a key way to improve their beneficial health effects and organoleptic and nutritional quality. Indeed, this approach of enriching the yogurt with phytoconstituents has gained significant interest, and now future investigations are being carried outin this direction.

In this sense, the present review discusses hypercholesterolemia and the clinical interventions used for its management. Moreover, it also discusses the health benefits of yogurt consumption and the concept of food enrichment. Additionally, it highlights the phyto-enrichment of yogurt and clinical evidence supporting its potential use in managing hypercholesterolemia.

## 2. Hypercholesterolemia

### 2.1. Low-Density Lipoprotein-Cholesterol and the Concept of Hypercholesterolemia

**Figure 1:**
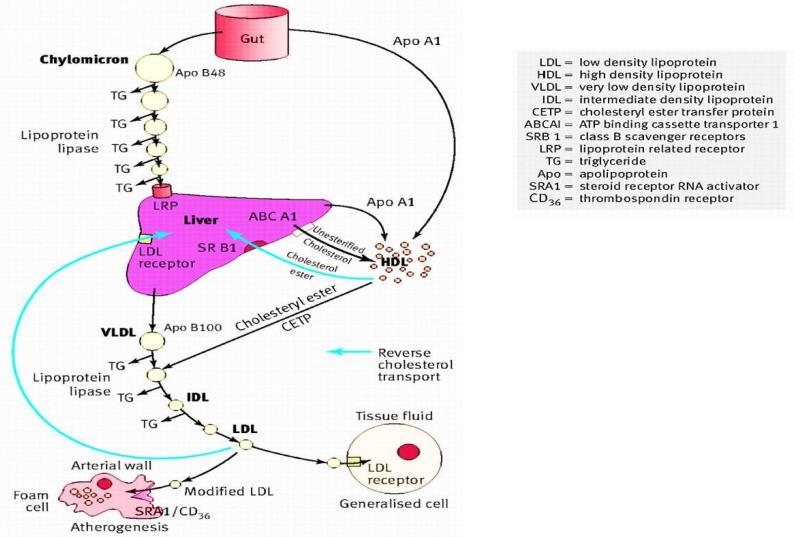
Diagrammatic representation of the cholesterol role in the lipoprotein metabolismadapted from [35] with license number 5090021099627.

Hypercholesterolemia (HC) is related to an elevated level of plasma low-density lipoprotein-cholesterol (LDL-C), as plasma LDL is the chief carrier of cholesterol [33]. The gut absorbs dietary cholesterol primarily packed in chylomicrons(triglyceride-rich); these chylomicrons first break down with the help of lipoprotein lipase (LPL), then the fatty acids and monoglycerides released are further supplied to the adipose tissue and muscles. In contrast, the remnant of chylomicron passes through the liver (Figure 1). Then liver produces very-low-density lipoproteins (VLDL), from which they are exuded into the bloodstream.VLDL becomes enlarged due to the presence of triglycerides in it. As these triglycerides are unable to accommodate themselves in VLDL, instead they start to aggregate themselves in the liver resulting in the development of fatty liver disease.LPL metabolizes VLDL and produces intermediate-density lipoproteins (IDL). Later, it becomesconverted to LDL. The liver also produces high-density lipoprotein (HDL) particles from cholesterol, incorporating cholesteryl esters released from peripheral tissues. In this way, HDL becomesinvolved in the reverse transport of cholesterol from peripheral tissues to the liver. Cholesteryl ester-laden HDL particles are moved to LDLs via cholesteryl ester transfer protein (CETP). Then, these cholesteryl esters are carted via LDL particles, which are absorbed by the liver and peripheral tissues (to a lesser extent). The LDL uptakes, as well as degradation in the liver, rely on LDL particles’ binding onto cell surface receptors (LDL-receptors) of hepatocytes. These receptors are particularly connected to apolipoprotein B (apoB) on the LDL particle. Then, the LDL: LDL-receptor complex is internalized via endocytosis. The whole internalization process is reconciled through the LDL receptor adaptor protein. These receptor molecules becomerecycled, and the LDL particles undergo lysosomal degradation. A protein expressed in hepatocytes, the proprotein convertase subtilisin/kexin type 9 (PCSK9), is believed to play a vital role in the catabolism of LDL-receptors [34].

From the genetic point of view, the genetic mutations encoding proteins are functional during LDL uptake, and catabolism (i.e., apolipoprotein-B (ApoB) via the APOB gene, PCSK9 protein via the PCSK9 gene, LDL-receptor via the LDLR gene and LDL receptor adaptor protein via the LDLRAP1 gene) and have been distinguished in causing familial HC due to degradation and defective LDL uptake, which causes a rise in the plasma LDL-C level, form the HC phenotype [33]. Extreme HC perfectly characterizes these conditions, with excessive LDL-C level elevation resulting in atherosclerosis and cardiovascular diseases [33]. In adults, HC is a profound trait generated by the interplay involving harsh factorssuch asobesity, excessive amounts of saturated fat-rich diets, physical inactivity, and the susceptible genotype [33].

### 2.2. Epidemiology

Among the Indian population, the prevalence of HC differs between rural (10–15%) and urban (25–30%) populations [36]. To date, different data-based studies were performed on the Indian population, including the India Migration Study (*n* = 1983), India Heart Watch (*n* = 6123), Indian Industrial Population Surveillance Study (*n* = 10,442), Indian Women Health Study (*n* = 4624), Indian Council of Medical Research (ICMR) Integrated Disease Surveillance Project (urban *n* = 15,223, slum/periurbann = 15,751, rural *n* = 13,517), a nationwide industry-sponsored FitHeart Study (*n* = 46,919) and the INDIAB study (*n* = 2042) [37,38,39,40,41,42]. Among those, Al-Kharj of Saudi Arabia showed a positive association between the prevalence of HC and increasing age in the general population [43]. Another study reported that overweight participants have a higher risk of suffering from HC. In addition, a publicly funded cross-sectional survey carried out on the French population found that the HC prevalence in France is 23.3%, while in Spain, a cross-sectional study conducted from June 2008 to October 2010 with 11554 representative individuals of the population aged ≥18 years found that 50.5% of the population suffer from HC [44]. In the elderly population of Cyprus, a study conducted found a positive association between smoking habits and HC, whereas an inverse connection was observed among HC, adherence to the Mediterranean diet, and an alcohol drinking, after controlling for sex, age, and other factors [45]. The different studies performed in various countries to assess the prevalence of HC are shown in Table 1.

### 2.3. Investigation and Laboratory Assessment

Currently, there are various therapeutic options to manage HC; however, before starting the treatment for HC, clinical examination, detailed history, and basic laboratory tests are needed to properly diagnose the causes of secondary HC, other complications related to atherosclerosis, and primary manifestations of lipoprotein changes [35]. In this sense, a family as well as occupational history, including the history of alcohol consumption, smoking, and dietary preferences, will help to determine the level to which these factors contribute to the risk of cardiovascular disease and/or HC and determine the patient’s ability to change lifestyle [35]. Regarding physical examination, it should include waist circumference, body weight, blood pressure, height, and a search for the presence of xanthoma [35]. Finally, family history is also an important factor in helping cascade testing at the primary stage of HC [55].

Moreover, during HC screening, it is advisable to obtain blood samples post-fasting for a minimum period of 10 h to avoid the postprandial contribution of plasma triglycerides [35]. If the plasma triglycerides range is in the limit of up to 4.5 mmol/L, the LDL cholesterol concentration can be calculated using the Friedewald formula:

Low density lipoprotein cholesterol = total serum cholesterol—(HDL cholesterol + (serum triglycerides/2.2)).

Non-HDL cholesterol = total cholesterol − HDL cholesterol.

Non-HDL cholesterol may act as a target for statin treatment if LDL-C cannot be measured owing to hypertriglyceridemia. The HDL cholesterol and total cholesterol: HDL ratio values help evaluate the risk of cardiovascular disease, but LDL cholesterol can be used as a therapeutic target whenever possible. Non-HDL cholesterol targets are 0.75 mmol/L more than the LDL cholesterol target. In addition, to assess the risk of cardiovascular disease, it is also important to determine fasting glucose concentration, and before starting lipid-lowering treatment, some parameters must be measured, such as creatine kinase activity, liver function tests, dipstick urine protein, and serum creatinine [35].

For routine assessment, many testing laboratories use handheld point-of-care testing (POCT) devices and automated analyzers depends on enzymatic assays a commercialised cholesterol quantitation assay kits [56,57]. Point-of-care testing (POCT) gives quick results, with simplefunction, making it more suitable for people screening tests. POCT clinical application has been found efficient in raising awareness regarding significance of lipid levels to prevent future stroke events and CVD [58]. The CardioChek PA analyzer (PTS Diagnostics) is a handy whole blood test system including a single test strip to determinetriglycerides (TG)HDL cholesterol (HDL-C) and total cholesterol (TC) [59]. In Brazil, the CardioChek PA analyzer used by health professional workers is well recommended for the proposed screening programs; the diagnostic performance is appropriate for use as part of national health services, providing quick and accurate results.

### 2.4. Treatment

#### 2.4.1. Non-Pharmacological Therapy

There are various non-pharmacological options to manage HC, and dietary therapy is one of the most important ones. Among others, this approach’s primary goals are to progressively lessen the intake of total fat, saturated fats (saturated fatty acids), cholesterol and attain desirable body weight. For example, it is advisable to reduce the intake of saturated fat daily up to 7% of calories, the daily intake of total fat to 25 to 35% of calories, to limit the dietary cholesterol quantity to no more than 200 mg/day; incite the intake of soluble fiber to 20 to 30 g/day, widely abundant in oats, beans, peas, and in some specific fruits [60]. Dietary fibers containing insoluble and soluble fibers attained from plants involvinglignin and non-digestible carbohydrates. Soluble fibers consist of viscous fibers such asmucilage, gum, β-glucans, pectin, fructans (inulin, fructooligosaccharides), and hemicellulose (non-viscous fibers). The insoluble fibers consist of some lignin, cellulose, and hemicellulose [61]. While both insoluble and soluble fibers are not digestible and can be fermented by bacteria with the help of enzymes to hydrolyze the fiber, soluble fibers are more easily fermented by the gut bacteria and have prebiotic functions providing a short-chain fatty acids source. As such, short-chain fatty acids are quickly absorbed by the large intestine, can be oxidized, and used for the production of energy. Short-chain fatty acids suchas propionic acid absorption have been found to reducethe synthesis of cholesterol in the liver, leading to lessening the blood cholesterol and enhanced water and sodium absorption into the colonic mucosal cells [62,63]. Alternatively, functional fibers are nondigestible carbohydrates that are either synthesized or extracted (isolated) and manufactured, to confer valuable health effects. Functional fibers are chitosan, β-glucans, chitins, cellulose, gums, fructans, resistant dextrins, lignin, polydextrose, pectin, and resistant starches, polyols, and psylliums [64].

There are numerous studiescarried outonmetabolism diseases to check the effect of oat β-glucan [65]. A meta-analysis that included 17 randomized controlled trials (RCTs) (916 hypercholesterolemic patients) displayed that consumption of β-glucan significantly reduced LDL-C (−0.21 mmol/L (8.1 mg/dL)) [66]. In a randomized, single-blind, wheat bran–controlled study, it revealed that within 8 h if 11 g oat bran β-glucantaken it nearly doubled the plasma secretion of bile acids and reduces serum cholesterol by measuring the metabolite 7-hydroxy-4-cholesten-3-one in the plasma [67].

It is also expected the inclusion of plant stanols or sterols in the diet (2 to 3 g daily) plus vegetable oils. Other target food items used in reducing total cholesterol include cold-water fishes, such as salmon, sardines, and mackerel, which have rich omega-3 fatty acid contents, widely recognized for lowering triglyceride levels. Similarly, soybeans present in soy, nuts, tofu, and various meat items have a potent antioxidant effect that can help in lowering LDL-C [60].

#### 2.4.2. Pharmacological Therapy

Among the various drugs available to manage HC, HMG-CoA reductase inhibitors, bile acid resins, fibric acids, niacin, and ezetimibe, are the most often used, as briefly described below.

##### HMG-CoA Reductase Inhibitors (Atorvastatin, Lovastatin, Simvastatin, Fluvastatin, Rosuvastatin, Pravastatin)

In de novo cholesterol biosynthesis, statins act by inhibiting the 3-hydroxy-3-methylglutaryl coenzyme A (HMG-CoA) reductase, disrupting the conversion of HMG-CoA into mevalonate, which is a rate-limiting step. From this activity, a reduction in LDL-C synthesis and enhancement in LDL-C catabolism mediated via LDL receptors emerge as the primary mechanisms for statins’ lipid-lowering effects [60,68].

##### Bile Acid Resins (Colesevelam, Colestipol, Cholestyramine)

The major action of bile acid resins (BARs) is to bind bile acids in the region of the intestinal lumen, with a concurrent interruption of enterohepatic circulation of bile acids, which then leads to a decrease in the pool size of bile acid and provokes the bile acids hepatic synthesis from cholesterol. Then, the hepatic pool of cholesterol depletion results in an enhancement in cholesterol biosynthesis and rising in LDL receptors number in the hepatocyte membrane, provoking and stimulating an increase in catabolism rate from plasma, which consequently reduces the LDL-C levels. High hepatic cholesterol biosynthesis may be paralleled by high hepatic VLDL production and, in such patients; BARs may exacerbate hypertriglyceridemia and hyperlipidemia [60,68].

##### Fibric Acids (Gemfibrozil, Fenofibrate, Clofibrate) and Niacin

Among the various fibric acids available, Gemfibrozil reduces the VLDL synthesis to a minimum level, with a simultaneous rise in the rate of apolipoprotein B elimination rich in triglycerides from plasma. Niacin (nicotinic acid) lowers the synthesis of hepatic VLDL, causing a reduction in LDL synthesis. In addition, by reducing its catabolism, niacin raises the HDL-C levels [56,57]. In general, Gemfibrozil or niacin is more effective than clofibrate in lowering VLDL-C production [60,68].

##### Ezetimibe

Ezetimibe restricts cholesterol absorption within the intestinal brush border; this is the unique mechanism that makes it superior for adjunctive therapy, supported as monotherapy, or used with statins. The daily dose given is 10 mg once, with or without food. It is worth mentioning that the use of Ezetimibe alone results in approx. 18% reduction in LDL cholesterol level, while when used in combination with statins, an LDL-C reduction of 12 to 20% is stated [60,68].

#### 2.4.3. Herbal Treatment

It is widely recognized that Mother Nature has a plethora of potential. With a broad spectrum of activity, a systematic review has suggested that medicinal plants are also rich sources of compounds with anti-HC action rather than saturated fat; plant sterols containing drinks and margarine or stanols can reduce the plasma cholesterol up to 10% [68].

## 3. Yogurt

### 3.1. Concept, History, and Health Effects

Yogurt (also spelled “yoghourt” or “yoghurt”) is a fermented milk-based product believed by some regulatory agencies worldwide as lactose-free and defined explicitly by containing *Streptococcus thermophilus* and *Lactobacillus bulgaricus* viable bacterial strains [69]. In ancient times, the use of yogurt for food purposes was recognized by other names over the millennia: cuajada (Spain), mast (Iran), katyk (Armenia), matsoni (Georgia, Russia, and Japan),roba (Sudan), zabadi (Egypt), laban (Iraq and Lebanon), dahi (India), coalhada (Portugal), leben raib (Saudi Arabia), iogurte (Brazil) and dovga (Azerbaijan). Indeed, it has been reported that milk-based products were added to the human diet near around 10,000–5000 BC, with the help of domestic milk-producing animals (such asgoats, cows, yaks, sheep, and camels, as well as buffalo and horses) [70].

**Figure 2:**
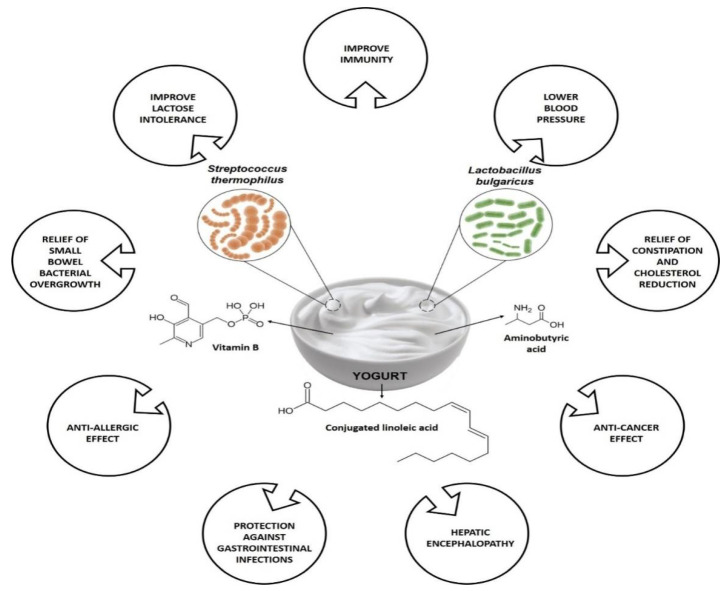
Variety of health benefits of yogurt consumption.

Indian Ayurveda dated back approx. 6000 BC, about the health-related benefits of having fermented milk-based items [69]. These days, around 700 different cheeses and yogurt items are consumed as part of Indian food. Yogurt was well known in the ROman and Greek empires, and the Greeks were the first to mention it in written references in 100 BC, noting the use of yogurt by barbarous nations [69]. In the book Bible, Abraham owed his long life and fecundity to consuming yogurt, as is referenced in the description of the “Land of Milk and Honey,” which several historians claim refers to yogurt [69]. Figure 2 summarizes some of the health benefits associated with yogurt consumption. Both yogurts’ bacterial and non-bacterial components are believed to play health-promoting effects in human health, specifically on the host’s immune system. Among other constituents, bioactive peptides, conjugated linoleic acid (CLA), group B vitamins, c-aminobutyric acid are a few metabolites are found in yogurt, and where the dairy products matrix, bioactive metabolites (i.e., exopolysaccharides and peptides) secreted throughout fermentation along with the active enzymes, fermentation process, are responsible for the health benefits of yogurt [71,72]. Based on fat content, the three most important types of yogurts currently available are low-fat, regular, and non-fat yogurts. Low-fat yogurts are made from partially skim or low-fat milk, whereas regular yogurt is formed from full-fat milk. Skim milk is also used for non-fat yogurt [73]. The nutritional composition of yogurt is shown in Table 2.

### 3.2. Concept of Food Enrichment

An enriched food refers to the food where nutrients have been added to supplement the product with nutrients that are usually present in the original form, but were removed during processing. For example, white bread is a commonly consumed enriched food where some specific vitamins are added because the bleaching process, depletes them [75].

In a broad sense, despite both enrichment and fortification refer to the addition of nutrients in food, the accurate definition varies. The WHO and the Food and Agricultural Organization of the United Nations (FAO) identifies the term fortification as “*the practicing deliberately increasing the essential micronutrient content, i.e., minerals (including trace elements) and vitamins in food irrespective of whether the nutritional components were primarily present before processing in food or not, so this is to enhance the quality of the nutrients food supply and to avail public health benefit with least health risk*”, whereas the term enrichment is defined as *“synonymous with fortification and refers to the adding micronutrients in food which are removed during the time of processing*” [75].

### 3.3. Phyto-Enrichment of Yogurt

Plants produce a broad pool of secondary metabolites that, among other functions, help them to adapt to various types of harsh environments, and defend them from different pests and microbial attacks, and resist all kinds of abiotic and biotic stresses. In this broad group of secondary metabolites, phenolic compounds have gained significant attention these days because of their anti-mutagenic, antioxidant, anti-clotting, and anti-inflammatory potential, which has been directly linked to their ability to decrease the risk of cardiovascular diseases and cancer development [76,77,78].

Phenolic compounds are the primary dietary source found in fruits [79]. It has been increasingly underlined that fruit juices, extracts, and powders show different biological activities, owing to which they have been used as functional ingredients in the food industry, such as dairy food industries [80,81]. Nonetheless, it is also worthy to note that the seasonal variation in vegetables and fruit production and the high demand for fresh fruits in the market have led researchers to explore alternative sources and strategies for the bioproduction of naturally-occurring bioactive compounds similar to phenolic acids and anthocyanins [82]. For example, plant callus andin vitro cell cultures were found to be promising tools for producing anthocyanins and other phenolic compounds, namely cherries, grapes, and carrots [82,83,84]. For instance, thesein vitro cultures have displayed various advantages over fresh fruit extracts in the possibility of producing natural compounds continuously, at a large scale depending on general needs, with less cost and opportunity of alternating the direction of phenolic or other anthocyanin’s biosynthesis [85,86].

In this way, several foodstuffs have also been used, and specifically, yogurt is a well renowned fermented dairy-based product. Despite its nutritional features and significance in the human diet, it is not being used as a chief source of phenolic compounds [87]. In dairy food products, the amount of phenolics is minimal due to cattle feed, including a low quantity of phenolics given, contamination of food production equipment with other sanitizing agents, and bacterial decomposition in milk proteins. Hence, some plant-derived additives have been incorporated to raise the phenolic content of yogurt, including the addition of four types of grape callus and grape varieties as functional ingredients [87,88]. Table 3 shows several studies addressing the impact of phyto-enriched yogurt on human health and general nutritional quality.

**Figure 3:**
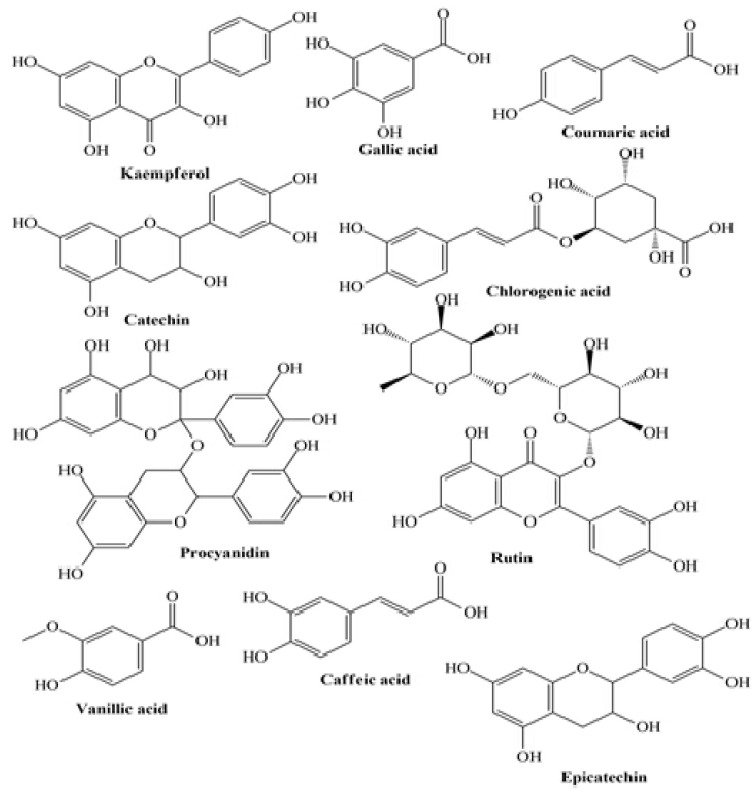
Some plant-derived phytocompounds present in enriched yogurt.

Jovanović et al. reported that apple pomace flour (APF)-enriched yogurts presented an increased total phenolic content and antioxidant activity in a dose-dependent manner [107]. Similarly, Jaster et al. revealed that anthocyanins present in strawberries-enriched yogurts were directly correlated with a higher antioxidant activity (ABTS) on the 1st and 7th day of storage for control and fortified yogurt, respectively [90]. A similar trend was stated using the DPPH method, indicating a strong correlation between the antioxidant activity and the anthocyanins content for control and fortified yogurts on the 1st and 7th day, respectively. Marand et al. revealed that after 14 days of storage, the total phenolic content in flaxseed enriched yogurt was higher than that of control yogurt, attributed to the phenolic compounds: ρ-coumaric acid, lignans, ferulic acid, and hydroxycinnamic acid derivatives [98] (Figure 3). The authors also showed that flaxseed-derived proteins are rich in sulfur amino acids (Met and Cys) and that the lignan protein joined with γ-tocopherol acts as a valuable, healthy compound, presenting good antioxidant activity. Helal and Tagliazucchi demonstrated in their study that in the supernatant of fortified yogurt, the addition of cinnamon powder led to a significant increase in the content of total phenolics as compared to the supernatant of plain yogurt [94]. The most valuable monomeric phenolic compounds present in the supernatant of cinnamon-fortified yogurt were cinnamaldehyde, identified and quantified by HPLC. Finally, Georgakouli et al. revealed that the intake of olive fruit polyphenol-enriched yogurt (400 g) with encapsulated olive polyphenols (experimental condition, EC; 50 mg) significantly reduced body mass index, body weight, hip circumference, and systolic blood pressure in nonsmoking volunteers when compared to plain yogurt (control condition, CC) after two weeks [103].

## 4. Phyto-Enriched Yogurt and Hypercholesterolemia Management: Clinical Evidence

There has been an increasing interest in addressing the impact of phyto-enriched yogurt in hypercholesterolemia management in clinical studies (Table 4). For example, Buyuktuncer et al., in a double-blind, placebo-controlled randomized study for 4 weeks, given to the control group (*n* = 35) a placebo yogurt and the intervention (*n* = 35) group consumed low-fat yogurt (115 g) with 1.9 g/day plant stanols as esters [112]. The main findings were a marked decrease in LDL cholesterol (6.3%), serum total cholesterol (4.6%), and non-HDL cholesterol (6.2%) levels from baseline in the intervention group as compared to the control. Insignificant changes were observed at the anthropometric level during the intervention. Similarly, Párraga-Martínez et al. conducted a double-blind, placebo-controlled study involving 182 adults diagnosed with HC [113]. The authors defined two study groups: the intervention group orally received 2 g of plant stanols containing yogurt drink (*n* = 91), and the control group received an un-supplemented yogurt (*n* = 91). At 12 months, the lipid profile, defined as the primary endpoint, was changed. In addition, the authors found a >10% reduction in LDL-C in the intervention group (RR 1.7; 95% CI 1.1–2.7). Similarly, Vásquez-Trespalacios et al. revealed in their study that the consumption of a yogurt drink (regular) supplemented with 4 g stanols (plant origin) added as esters compared to yogurt drink (Benecol^®^, Colanta) promoted a significant reduction in LDL-C and total cholesterol [114]. Doornbos et al. revealed that as compared to a placebo, a single-dose yogurt drink (100 g) taken with a meal regardless of its fat content led to a marked decrease in LDL-C [115].

## 5. Conclusions

Yogurt is proclaimed as a healthy fermented milk-derived product containing rich contents of protein of high quality. Several studies have underlined that yogurt has a beneficial effect on individuals suffering from HC due to the presence of conjugated linolenic acid, calcium, casein, probiotics, and proteins. As per the available data, various factors, including the amount of yogurt consumed by the individual under evaluation, fat content, type of milk, and difference among targeted populations, act as direct contributors to the health beneficial effects of yogurt. Additionally, yogurt has been stated to significantly improve the quality of diet and overpower appetite, ultimately helping to regulate glucose metabolism and control obesity. Moreover, supplementation of dietary fiber has been reported to influence the quality parameters of yogurt as they act as bulking agent, colorant, fat replacer, functional ingredient, nutraceutical and probiotic agent. Furthermore, incorporation of dietary fibers has been proclaimed to enhance satiety more effectually in contrast to other beverages. Owing to which, the incorporation of dietary fiber in diet and control intake of food is effective way to regulate the cholesterol level.Despite the low cholesterol levels and saturated fatty acids, yogurt overcomes the negative aspects via its nutrient-rich compositions encompassing various minerals and vitamins that play a substantial role in preventing and treating HC-induced complications.

Nonetheless, yogurt processing degrades its content of minerals, vitamins, and other vital constituents, which have a beneficial effect on an individual’s health. Thus, the overlooked option of enriching yogurt with phytoconstituents has attracted considerable attention. Differentin vitroand clinical studies have provided new insights into the benefits of phytoconstituents for improving gut microbiota and human health. Specifically, studies related to the use of phyto-enriched yogurt have unveiled that it effectively prevents HC and other chronic diseases. Taken together, the data discussed here underline that the phyto-enrichment of yogurt has opened and will even boost new avenues for food sector industries that could serve as an effective and healthy alternative for treating HC, instead of conventional treatment approaches.

## Figures and Tables

**Table 1:** Epidemiological studies related to the prevalence of hypercholesterolemia.

Study Country	Year Reported	Sample Size	Prevalence (%)		Reference
			Men	Women	
Spain	2008–2010	11,554	25.5	26.4	[4]
Kazakhstan	2012–2015	954	28.4	44.1	[9]
India	2014	6123	25.1	24.9	[41]
Saudi Arabia	2016	1019	56.7	43.3	[43]
France	2014–2016	2321	27.8	19.0	[44]
Cyprus	2004–2005	150	60	68	[45]
Kingdom of Bahrain	2012	166	3	12.5	[46]
Thailand	2004	39,290	14	17	[47]
Mexico	1987–1988	33,588	10.6	10.6	[48]
Iran	2016	21,293	23.8	29.8	[49]
United States	2015–2016	ND	28.5	8.9	[50]
Japan	2010	2417	21.5	31	[51]
Bangladesh	2006	2610	2.2	0.5	[52]
Malaysia	2015	19,935	43.5	52.2	[53]
Kuwait	1995	1076	16	15.7	[54]

ND—Not defined.

**Table 2:** Composition of nutrients in yogurt with different fat content [74].

Nutrients (per 100 g)	Whole Milk Yogurt	Low-Fat Yogurt	Non-Fat Yogurt
Energy (kcal)	61	63	56
Water (g)	87.89	85.06	85.22
Protein (g)	3.47	5.25	5.73
Fat total (g)	3.25	1.55	0.18
Sugar total (g)	4.66	7.04	7.68
Saturated fatty acids (g)	2.096	1	0.116
Monounsaturated fatty acids (g)	0.893	0.426	0.049
Polyunsaturated fatty acids (g)	0.092	0.044	0.005
Cholesterol (mg)	13	6	2
Carbohydrate (g)	4.66	7.04	7.68
Sodium (mg)	46	70	77
Vitamin A, RAE (µg)	27	14	2
Thiamin (mg)	0.029	0.044	0.048
Riboflavin (mg)	0.142	0.214	0.234
Niacin (mg)	0.08	0.114	0.124
Folate (µg)	7.0	11	12
Vitamin B_12_ (µg)	0.37	0.56	0.61
Vitamin K (µg)	0.2	0.2	0.2
Magnesium (mg)	12	17	19
Calcium (mg)	121	183	199
Iron (mg)	0.05	0.08	0.09
Phosphorus (mg)	95	114	157
Potassium (mg)	155	234	255
Zinc (mg)	0.59	0.89	0.97
Copper (mg)	0.009	0.013	0.015

RAE—Retinol activity equivalents.

**Table 3:** Variety of phytoenriched yogurt and their effects on nutritional quality and general health.

Study Country	Chemical Composition and Physical Nature	Plant Name	Part Used	Quality Effects	Health Effects	Reference
Australia	ND/Set type	Pineapple (*Ananas comosus* L. Merrill)	Peel and pomace powder	Increase in probiotic population by 0.3–1.4 log cycle	Remarkable antioxidant activity in case of DPPH, (IC_50_ = 0.37–0.19 mg/mL) and hydroxyl radicals (58.52–73.55%)	[89]
Brazil	Full-fat milk/Set type	Strawberry (*Fragaria* × *ananassa*)	Juice	Higher total lactic acid bacteria count, i.e., 10^8^ CFU/mL; Decrease in viscosity	Three-fold increase in total anthocyanins content; Antioxidant activity in DPPH was 8.86–9.19 mgGAE/mL and in ABTS it was 0.26–0.38 mgGAE/mL	[90]
Egypt	Full-fat milk/Set type	Green tea (*Camellia sinensis* L.) and *Moringa oleifera*	Leaves extract	No suppression of starter culture growth; No significant change in viscosity; green tea extract improved consistency	High total phenolic content in case of green tea extract (712 mgGAE/100 g) and *Moringa* (280 mgGAE/100 g); DPPH radical scavenging activity in case of green tea extract (LC_50_ = 64 µg/mL) and in *Moringa* (LC_50_ = 65 µg/mL)	[91]
Saudi Arabia	Full-fat milk/Set type	Fenugreek (*Trigonella foenum-graecum*) and *Moringa oleifera*	Seed extract	Increase in the viable count of *Streptococcus thermophiles* and *Lactobacillus delbrueckii* subsp. *bulgaricus*	Increase in total phenolic content and antioxidant activity; Increase in mineral content viz. Ca, P, K, Mg, Zn, and Fe; Antibacterial activity against *Escherichia coli*, *Staphylococcus aureus*, *Lesteriamonocytogenes*, and *Salmonella* spp.	[92]
Italy	Full-fat milk/Stirred	Hazelnut (*Corylus avellana* L.)	Skin	Increase the viable count of *Streptococcus thermophiles* (8.38 log_10_CFU/mL) and *Lactobacillus delbrueckii* subsp. *bulgaricus* (7.73 Log_10_CFU/mL)	Increase in total phenolic content (13.12–19.43 µgGAE/g) and an increase in antioxidant activity in DPPH (25.27–47.29 TEµM/g)	[93]
Egypt	Full-fat milk/Stirred	Cinnamon (*Cinnamomum cassia*)	Bark powder	Nd	Increase in total phenolic content (28.3 mg catechin/100 g); Fortified yogurt exhibited significantly higher radical scavenging activity than the plain yogurt both in the ABTS and DPPH assay ( *p* < 0.05)	[94]
Tunisia	Low-fat milk/Set type	Pomegranate (*Punica granatum* L.)	Seeds	Change in color; Decrease in firmness	Increase in antioxidant activity; Increase in acceptance (based on sensory)	[95]
South Korea	Low-fat milk/Stirred	Lotus (*Nelumbo nucifera*)	Leaf powder	No significant change in the viability of lactic acid bacteria; 4-fold increase in viscosity	Increase in total phenolic content (47.94–61.94 µgGAE/g) and DPPH activity (48.81–52.34%)	[96]
Saudi Arabia	Low-fat milk/Set type	Argel (*Solenostemma argel* Hayne)	Leaf extract	Increase in acidity, lactic acid bacteria count, water holding capacity, viscosity, and stability	High total phenolic content (23.79 mgGAE/100 g) and DPPH activity (36.39%)	[97]
Iran	Full-fat milk/Stirred	Flaxseed (*Linumusitatissimum* L.)	Powder	Increase in acidity, water holding capacity, and viscosity	Increase in DPPH scavenging capacity (45.83%)	[98]
South Korea	Low-fat milk/ND	Aronia (*Aronia melanocarpa*)	Juice	Increase in lactic acid bacteria count (9.59 logCFU/mL); Increase in total acidity	Increase in DPPH scavenging capacity (77.87%), ABTS (70.90%) and reducing power (29.86%); Increase in total phenolic content (54.05 mgGAE/g) and total flavonoids (152.10 mgCatechin/g)	[99]
Mexico	ND	Red cactus pear (*Opuntia ficus-indica* L.)	Peel and mucilage powder	Magenta color produced	Increase in total phenolic compounds, total flavonoids, total betalains, inhibition capacity, and reducing power, respectively	[100]
Indonesia	Full fat milk/ND	Roselle (*Hibiscus sabdariffa* L.)	Flower extract	Increase in viscosity, holding capacity; Decrease in total lactic acid bacteria count	Increase in DPPH scavenging capacity	[101]
South Korea	Low-fat milk/ND	Olive (*Olea europaea*)	Powder	Decrease in viscosity and total lactic acid bacteria count	At day zero storage, the total phenolic content (6.96 mgGAE/100 g), DPPH scavenging capacity (47.53%), and reducing power (0.57%)	[102]
Greece	Low-fat milk/ND	Olive (*Olea europaea*)	Polyphenols	Increase in lactic acid bacteria count	Decreased levels of low-density lipoprotein (LDL) cholesterol and thiobarbituric acid reactive substances	[103]
South Korea	Non-fat milk/ Set type	Green tea(*Camellia sinensis* L.)	Powder	Increase in lactic acid bacteria count	Decreased expression of TNF-α and IL-1β in a human colorectal cell line, HT-29	[104]
Malaysia	Full-fat milk/ND	Neem (*Azadirachta indica*)	Leaves powder	Nd	Increase in total phenolic content (74.9–19 µgGAE/mL) and increase in antioxidant activity in DPPH (53.1%); Increase in enzymes inhibition (α-amylase 47.4%, α-glucosidase 15.2% and angiotensin-1 converting enzyme 48.4)	[105]
Italy	Full-fat milk/ND	Grape (*Vitis vinifera*)	Skin powder	Decrease in syneresis and fat; Increase in acidity	Increase in total phenolic content, and antioxidant activity	[106]
Serbia	Full-fat milk/Set type	Apple (*Malus domestica*)	Pomace flour	Increase the firmness and viscosity	Increase in total phenolic content, radical scavenging (DPPH), and reducing activity (FRAP); Inhibit colon cancer cells’ viability	[107]
Nepal	Full-fat milk/Set type	Mulberry (*Morus* L.)	Osmo- dried fruit	Reduce in syneresis	Increase in total phenolic content (68.03 mgGAE/100 g), an increase in anthocyanins content (7.9 mg/100 g), and an increase in antioxidant activity in DPPH (47.6%)	[108]
Saudi Arabia	Full-fat milk/ND	Nutmeg (*Myristica fragrans* Houtt.), Black pepper and white pepper (*Piper nigrum* L.)	Water extract	High production of lactic acid	Radical scavenging activity was positively affected; High total phenolic content	[109]
Egypt	Full- fat milk/ND	Watermelon (*Citrullus lanatus*)	Seed milk	Apperance, flavor, body and texture, and overall acceptability was best with 50% cow’s milk and 50% watermelon seed milk	Improved the renal function in hyperuricemic rats; Enhancment of the activities of superoxide dismutase, catalase, and glutathione transferase	[110]
Egypt	Whole milk powder/	Fennel (*Foeniculum vulgare*)	Seed water extract	Titratable acidity significantly decreased	Antioxidant activity significantly increased; Total phenolic content increased	[111]

ND—Not defined; Nd—Not done; CfU—Colony-forming unit; DPPH-2,2-diphenyl-1-picrylhydrazyl; LC_50_—Lethal concentration 50%; GAE—Gallic acid; ABTS-2,2’-azino-bis(3-ethylbenzothiazoline-6-sulfonic acid; TE—Trolox equivalent; TNF-α-Tumor necrosis factor alpha; FRAP—Ferric ion reducing antioxidant power.

**Table 4:** Clinical trial studies evaluating the effect of phyto-enriched yogurt on hypercholesterolemic subjects.

Study County	Type of Study	Sample Size (n)/ Mean Age (Years)	Characteristics of the Subject	Dosage Concentration	Period of Intervention	Effect	References
Italy	Retrospective	24/52 ± 12	BMI (kg/m^2^): 27.3 ± 2; hypercholesterolemia	Sterol enriched yogurt (1.6 g/day)	48 days	↓ LDL (23 ± 4 mg/dL)	[116]
Turkey	Randomized	35/45.5	BMI (kg/m^2^): 27.9 ± 3.15; untreated mild to moderate hypercholesterolemia	Sterol enriched (1/9 g/day) low-fat yogurt(115 g/day)	4 weeks	↓Serum total cholesterol (4.6%) ↓LDL (6.3%)	[112]
Spain	Randomized	91/54.8	BMI (kg/m^2^): 28.3; hypercholesterolemia	Stanol enriched (2 g/day) yogurt	12 months	↓ LDL (13.7 mg/dL)	[113]
Colombia	Randomized	40/37.9	BMI (kg/m^2^): 25.0; moderate hypercholesterolemia	Stanol enriched yogurt 4 g (2 pots/day)	4 weeks	↓ Serum total cholesterol (7.2%) ↓LDL (10.3%)	[114]
Italy	Randomized	30/ND	BMI (kg/m^2^): 24.6; moderate hypercholesterolemia	Sterol enriched (1–2 g/day) low-fat low-lactose yogurt	8 weeks	↓ LDL	[117]
India	ND	48/ND	BMI (kg/m^2^): ND; mild hypercholesterolemia	Sterol enriched yogurt (200 g/day)	30 days	↓ Serum total cholesterol (4.3%) ↓LDL (5.3%)	[118]
South Korea	Randomized	51/28.5	BMI (kg/m^2^): 22.8; mild hypercholesterolemia	Stenol enriched (2 g/day) 150 mL strawberry yogurt	4 weeks	↓ Serum total cholesterol (6%) ↓LDL (10%)	[119]
Australia	Randomized	42/60.4	BMI (kg/m^2^): 26.5; moderate hypercholesterolemia	Sterol enriched (1.8 g/day) and stanol enriched (1.7 g/day) low-fat yogurt (300 g/day)	3 weeks	↓ LDL (6%) ↓ LDL (5%)	[120]
Netherlands	Randomized	184/57	BMI (kg/m^2^): 25.2; moderate hypercholesterolemia	Sterol enriched (3 g/day) yogurt (100 g/day)	4 weeks	↓ LDL (9.3–9.5%)	[115]
Canada	Randomized	26/59.6	BMI (kg/m^2^): 26.4; hypercholesterolemia	Sterol enriched (1.6 g/day) low-fat yogurt	4 weeks	↓ LDL (8.69%)	[121]

ND—Not defined; BMI—Body mass index; ↓— decreased.
